# Socioeconomic status influenced dispersal in early adulthood in Finland from 1760 to 1969

**DOI:** 10.1016/j.isci.2026.115467

**Published:** 2026-03-25

**Authors:** Jenni J. Kauppi, Alyona Artamonova, Milla Salonen, Mirkka Lahdenperä, Virpi Lummaa

**Affiliations:** 1 Department of Biology, University of Turku, Turku, Finland; 2 Population Research Institute, Väestöliitto, The Family Federation of Finland, Helsinki, Finland

**Keywords:** Human geography, Social sciences, Economics, Sociology

## Abstract

Dispersal away from the place of birth shapes an individual’s life course and has effects on the demography of populations. Parental socioeconomic status (SES) might shape dispersal decisions of young individuals by providing resources that enable dispersal or philopatry. High familial wealth can allow young adults to remain in their birth place or, in contrast, provide necessary resources to disperse. Using a large demographic dataset from Finland (1760–1969), we examined how parental SES influenced both the probability and distance of dispersal among young men and women over time. Individuals from high-SES families were more likely to remain in their birth parishes than those from low-SES background. Across the study period, both the likelihood and distance of dispersal increased, reflecting the broader societal transitions. Our findings highlight how socioeconomic resources and historical changes impact dispersal behavior, revealing disparities in how such changes affect young adults with differential access to parental resources.

## Highlights

• Overall dispersal distances increased over time for young men and women
• Young adults with low socioeconomic status always had high dispersal probabilities
• Individuals from higher-SES families had steep increase in dispersal over time

## Graphical abstract

**Figure:**
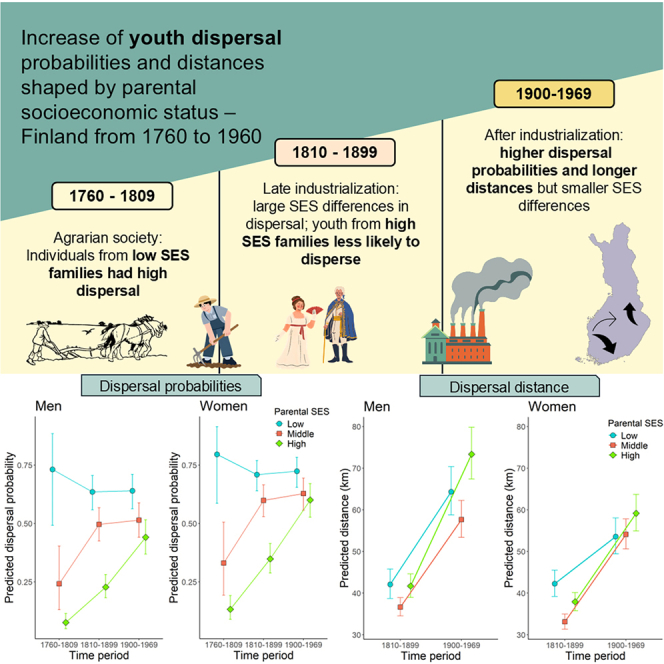

## Introduction

Dispersal—defined here as the movement of an individual from one location to another, with potential consequences for gene flow between populations^1^—shapes an individual’s life course and fitness outcomes and has downstream effects on demography and genetic structure of populations.^1^^,^^2^ Similar to other species, humans’ dispersal behavior varies considerably between and within populations. Humans disperse for several proximate reasons including, but not limited to, marriage (mate selection), education, or for work opportunities (resources), and avoiding competition or increasing cooperation with kin or other individuals.^3^^,^^4^^,^^5^^,^^6^^,^^7^ Philopatry is the counterpart of dispersal, and it can be used to refer to the individuals who stay in their natal (birth) areas, usually to reproduce.^8^ Internal migration is another well-established concept describing within-country movements of humans, and its underlying economic, social, environmental, and political factors are closely aligned to what influences dispersal in humans. However, dispersal is a more specific term, explaining the spreading of species from their birth location to a new area, which is often a one-time event, while internal migration is a human movement within a country that can be undertaken at different life course stages. In this study, we use the term “dispersal” but utilize useful studies on internal migration and, specifically, the first independent moves of individuals away from parental home.

Multiple studies have found that the propensity to disperse in humans is highest at the ages from around teen to late twenties.^6^^,^^9^^,^^10^ Dispersal of young adults differs from that of other age groups as it takes place during a phase marked by significant life changes related to physical maturation, as well as cognitive, social, and interpersonal developments.^11^ Despite differences from the other age groups, dispersal of young adults can be discussed from the perspective of general theoretical models of internal migration. The push and pull theory^12^ claims that migration is determined by the forces that induce people to move to a new geographical location. Push factors are the conditions that can force people to leave their homes and are related to the place from which a person migrates (e.g., non-availability of opportunities, poverty, and loss of wealth), while pull factors draw people toward certain locations because of the potential benefits at the new place (e.g., better job opportunities, working and living conditions, availability of land for agriculture, and better education facilities). Neoclassical economic models of migration, in turn, view migration as an investment decision made by rational individuals who can estimate costs and returns of migration. Migration occurs when the expected returns (for example, higher lifetime earnings) exceed the costs of moving.^13^^,^^14^ Most of the theories considering migration are mainly based on the optimization strategy of individuals or families to balance the costs and benefits of migration^15^ which are not unlike the discussed costs (e.g., loss of familiarity to environment or sociality)^16^ and benefits (e.g., better resources and avoiding competition with kin)^2^ of dispersal.

The life course perspective^17^ links leaving birth place with life events marking the transition to adulthood, including the initiation of a working trajectory, securing livelihood, entering marriage, and obtaining education, all of which can endorse dispersal.^9^^,^^18^^,^^19^^,^^20^ Alternatively, staying in the birth place (philopatry) might be beneficial, mostly because of location-specific capital,^21^ i.e., familiarity with the birth place environment and other individuals; staying can even increase investment in kin or cooperation with them.^22^^,^^23^^,^^24^ Individual-level dispersal behaviors, including the likelihood of dispersal and the distance traveled, can be affected by environmental factors (e.g., climate change and war) and social factors (e.g., closeness to kin), individual characteristics (e.g., sex and age), or available resources (e.g., wealth, territory, and food).^4^^,^^5^^,^^7^^,^^25^^,^^26^^,^^27^^,^^28^^,^^29^ In particular, research suggests that familial resources are an important factor affecting dispersal behavior.^5^^,^^7^^,^^11^^,^^30^

Family wealth might either allow young people to stay in their birth place or provide necessary resources for dispersal^31^; lack of wealth might prevent dispersal if it has high costs^29^^,^^32^ or force individuals to move elsewhere, as voluntary dispersal is often undertaken in search for better economic opportunities.^30^^,^^33^ Numerous studies have suggested that the socioeconomic status (SES) of the family of origin plays a crucial role in dispersal, both historically and in contemporary times, albeit through varying mechanisms.^7^^,^^11^^,^^30^^,^^31^^,^^33^ For European societies of the past, higher socioeconomic position—landownership— often tied people to a place.^34^ Farmers were found to remain in the family farm all their lives, especially eldest sons of a family, as they were often the first in line to inherit the family farm,^9^^,^^28^^,^^35^^,^^36^^,^^37^ whereas children from laboring and middle-class families left their birth place either to work as farmhands or to become domestic servants.^34^ Additionally, a study based on data from 1850 to 1913 in Norway found that higher parental wealth discouraged migration, both internal and international, compared with poorer families.^37^ Alternatively, parental income might serve as a significant material resource, which could help young adults to cover expenses associated with migration or establishing a new household.^11^^,^^38^ Parental level of education is a modern indicator of family resources that relates to migration; young adults whose parents are highly educated have a greater tendency to move to pursue higher education.^39^

Many Western societies have traditionally been patrilocal, meaning that paternal kin commonly lived nearby or in a same household with each other,^36^ and wealth was passed on through male lineage, and, therefore, dispersal was often female biased. Young women were more likely to move from the birth place in general and more likely than men to move for marriage, suggesting that brides commonly moved to their future husband’s home, whereas men were more likely than women to move for work-related reasons.^7^^,^^34^ However, a study^9^ from the UK between 1750 and 1994 found that the reasons for men’s and women’s dispersals have changed over time. After industrialization, from the mid-nineteenth century onward, women started to move for employment almost as often as men,^9^ perhaps reflecting the changing role of women in society.^40^ A study from twentieth-century America points to similar trends.^41^ However, women were still more likely to move or leave home for marriage than men at least in post-war Sweden.^42^ Importantly, it seems that moving long distances (versus moving from the birth place but staying in the home region) was, historically, a male strategy, which is also related to the search for employment,^43^ indicating that women would move shorter distances because of marriage-related reasons.

Large-scale shifts in society, i.e., in industrialization, urbanization, growing economy, and demographic transition, can change dispersal behavior of individuals in a population. The frequency of dispersal has been found to increase in time, and the dispersal distances became greater after industrialization in many countries.^44^^,^^45^^,^^46^^,^^47^^,^^48^ In historical times (time before industrialization in European societies), the intensity of dispersal and the moving distance are assumed to be affected by family wealth, inheritance customs, household structures, marriage patterns, and ties between kin.^49^ Skilled workers and individuals from families of higher or middle occupational class tended to move longer distances from the birth place than unskilled workers, while the latter were more likely to move within the local labor market area.^9^^,^^50^ After industrialization, changes in these customs also shaped the overall dispersal behavior. Nowadays, pursuing education is a prominent reason for dispersal,^20^ especially long-distance migration among young adults that requires greater family resources.^51^^,^^52^ What unites the dispersal behavior nowadays and in the past is the importance of family resources available to young people, which is reflected in familial SES. Another potential factor for change that is perhaps closely linked to familial SES is familial obligation. In the 19th century and well into the early 20th century, familial obligations and controls were dominant in influencing transitions to adulthood, including leaving parental home, but the post-war era brought about changes from familial to non-familial dominance.^3^

Exploring dispersal behavior and its determinants over time is critical for studies on life-history maximizing strategies (reproducing and survival of offspring),^28^ as well as for understanding the demographic and social transitions.^53^ However, historical studies of the moves of young men and women from families with unequal access to resources in successive birth cohorts are relatively rare and focus on a limited number of countries (see Falkingham et al.^40^ and Pooley et al.^9^ for the UK; Kok et al.,^34^ Paping,^54^ and Sesma Carlos^50^ for the Netherlands; and Beise et al.^7^ for Germany). Additionally, relatively little research has been done on the distance young adults move, even for the recent periods (for example, see Pooley et al.,^9^ Leopold et al.,^55^ and Gillespie et al.^52^). The main reason for the scarcity of research on this topic and its geographic bias is the limited availability of high-quality historical data.

The aim of this study is to explore how natal SES (parental SES) relates to the probability of dispersal, here defined as leaving the birth place (out-of-birth parish moves) and the dispersal distance, of young men and women between ages 15 and 35 years. We also focus on the changes in this relationship over time in Finland from 1760 to 1969 as the society underwent demographic transition, urbanization, industrialization, shift from multigenerational patrilocal families to nuclear families, and other major societal transformations. By comparing men’s and women’s probability to disperse and dispersal distances in three different periods in Finland (1760–1809, 1810–1899, and 1900–1969) and among representatives of differing natal SES (SES groups: low [e.g., servants and dependent lodgers], middle [e.g., tenant farmers, craftsmen, and small-land farmers], and high [e.g., land owners and clergy]), we assess how an individual’s access to resources at young ages shapes dispersal patterns in different contexts. We fit the generalized linear mixed model with binomial outcome of the out-of-parish moves (dispersal = yes/no), explained by the SES, sex, and time, and we control for regional variation and parental clustering in a sample of 22,429 individuals. In another model, we fit the linear mixed-effect model with the response of dispersal distance (distance between the parish of origin and the first dispersal parish), explained by the same variables as in the first mentioned model but without control for regional variation, in a sample of 8,439 individuals.

Finnish society provides an interesting case for exploring the relationship between natal SES and dispersal over time. Finland has been keeping records of people’s lives in church registers—collected by the Lutheran Church in parishes—continuously since the 18^th^ century, and by 1749 onwards, it was legally required in the whole country.^56^ Historically, Finland was a poor agricultural and patriarchal society under the Swedish rule (1721–1809) and Russian rule (1809–1917)^57^ before it gained independence in 1917. The country had large gaps between the wealthy and the poor in the 19th century; most differences were observed between the estates and those outside any estate, and wealth was a determining factor for the status and value of individuals.^58^^,^^59^ Finland had late urbanization and industrialization compared with other European countries; before the beginning of the 20^th^ century, 80% of the population was still active in the agrarian practices and lived in the countryside.^35^^,^^60^ Additionally, at the end of the 1800s, there were laws that limited moving between parishes and prevented changing professions, impacting the possibilities of dispersal.^59^ Exploring this topic in Finland will enrich our understanding of historical human dispersal that was based mainly on data from the UK, the Netherlands, America, and Canada where urbanization and industrialization unfolded differently.

Following the literature on dispersal, we test several hypotheses.

H1) Dispersal probability and dispersal distances increase over time, for all parental SES groups and both men and women.
H2) Familial resources (SES) tend to affect dispersal probability and dispersal distance differently in different time periods.
  a) Before industrialization: Individuals from low-SES families are more likely to disperse but move shorter distances than the representatives of the middle- and high-SES families.
  b) After industrialization: Individuals from the high- and middle-SES families are as likely to move from the birth place and disperse similar distances as the representatives of low-SES families.
H3) Women might be more likely to disperse than men, but men tend to move farther than women.

## Results

### Dispersal probabilities

**Figure 1:**
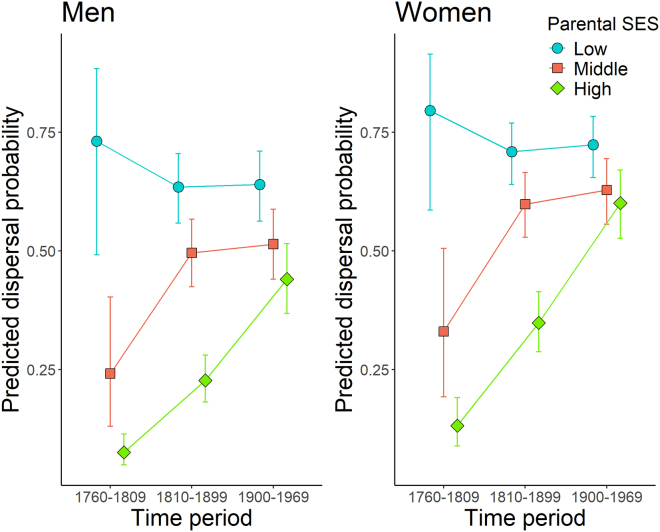
The predicted probabilities of dispersal by the parental SES group and sex over time A two-way interaction of time and family SES group is shown, separately for men and women, as women have often higher dispersal probabilities than men. Each parental SES group is represented by their own color and shape with error bars (±95% confidence interval). The lines between the same SES group represent the change from one time period to another.

Overall, dispersal probability changed through time and varied between different parental SES groups and sexes. The highest dispersal probabilities were observed in the latest time period; low- and middle-SES groups had the smallest change through time, and they dispersed, in most cases, more than the high-SES group, with women having higher dispersal probabilities than men (Figure 1, *n* = 22,429). We found statistically significant interaction effects between parental SES and time period (χ^2^ = 88.43, df = 4, and *p* value < 0.0001; Table S1) and between parental SES and sex (χ^2^ = 7.47, df = 2, and *p* value = 0.0237; Table S1), but we found no statistically significant interaction between sex and time period (χ ^2^ = 0.39, df = 2, and *p* value = 0.8231; Table S1). The interactions show that the intensity of the difference in dispersal probability between men and women varied in each parental SES group (Figure 1). Women were more likely to disperse than men across the observed time periods, but the difference between the sexes varied depending on the parental SES group. Within the low-parental SES group, odds of dispersing were 42% higher for women than for men in the same group (odds ratio = 1.42, SE = 0.163, z ratio = 3.068, and *p* value = 0.0022), and within the high-parental SES group, women’s odds of dispersing were 86% higher than those for men (odds ratio = 1.86, SE = 0.156, z ratio = 7.34, and *p* value <0.0001). Women from the middle-parental SES group had 54% higher odds of dispersal than men from the same group (odds ratio = 1.54, SE = 0.143, z ratio = 4.67, and *p* value = <0.0001). These differences did not change significantly through time, as there was no statistically significant interaction between sex and time period in our model (Table S1).

Parental SES had a statistically significant interaction with the time period variable, similarly for both men and women (Table S1), indicating that dispersal behavior changed within and between the SES groups over time (Figure 1). Comparison between the parental SES groups showed that individuals in the low-SES group had the highest probability to move in each time period than those in the other SES groups. For example, in the 1900–1969 time period, men from low-SES families had 2.26 higher odds of dispersal than men from high-SES families, and women from low-SES families had 1.74 higher odds of dispersal than women from high-SES families (Table S2). The middle-SES group had lower dispersal probabilities than the low-SES group for men and women similarly in all time periods, and in the 1760–1809 and 1810–1899 periods, the probability of dispersal for middle-SES group was significantly higher than that for the high-SES group (Figure 1 and Table S2). However, in the period 1900–1969, middle-SES women were not more likely to disperse than high-SES women, but for men, there was a statistically significant difference between the groups (Figure 1 and Table S2). All odds ratios of dispersal probabilities between the parental SES groups are presented in Table S2.

The probability to disperse was stable over time for both low-SES men and women (Figure 1 and Table S3). The high-SES group had the strongest increase in their dispersal probability through time, similarly for men and women. For example, the odds of dispersal in 1900–1969 compared with that in 1760–1809 were 9.58 times higher for men and 9.99 times higher for women (Figure 1 and Table S3). The middle-SES group exhibited an increase in their dispersal probability from 1760–1809 to 1810–1899, after which the probability of dispersal remained stable from 1810–1899 to 1900–1969 (Figure 1 and Table S3). All odds ratios of dispersal probabilities between the three time periods are presented in Table S3.

### Dispersal distance

**Figure 2:**
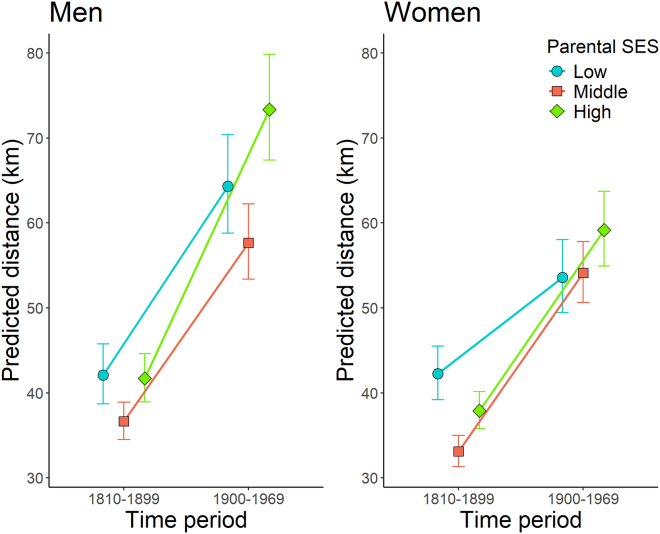
The predicted dispersal distances by the parental SES group and sex over time A three-way interaction of sex, time, and family SES group is shown, with those for men and women shown separately for clarity. Each parental SES group is represented by their own color and shape with error bars (±95% confidence interval). The lines between the same SES group represent the change in dispersal distance from one time period to another.

Overall, men had, in most cases, longer dispersal distances than women in both time periods (1760–1809 time period was excluded due to a small sample size) and all SES groups (Figure 2; *n* = 8,439). Men and women differed in their moving distances depending on parental SES, and this difference changed through time, as suggested by a statistically significant three-way interaction between sex, SES, and time period (χ^2^ = 6.88, df = 2, and *p* value = 0.0321; Table S4). First, by comparing men and women within the same SES groups, we found that men moved, on average, farther away from the birth place than women in most SES groups and in both time periods (Figure 2 and Table S5). There were exceptions: men and women had similar dispersal distances in the low-parental SES group in the 1810–1899 time period (z ratio = −0.074, SE = 0.049, and *p* value = 0.9413; Table S5), and there was no statistically significant difference between men and women in the middle-SES group in the 1900–1969 time period (z ratio = 1.066, SE = 0.047, and *p* value = 0.1522; Figure 2 and Table S5). The largest difference was between the high-SES group men and women in 1900–1969, when, for men, the predicted average moving distance was 73.3 km and equaled 64.3 km for women (z ratio = 4.455, SE = 0.060, and *p* value < 0.0001; Figure 2 and Table S5). All pairwise comparisons in dispersal distance between men and women are presented in Table S5.

Second, there were also differences in moving distances between the SES groups within each time period, as well as between men and women (Figure 2 and Table S6). All pairwise differences of the dispersal distances between SES groups in both time periods are presented in Table S6. In 1810–1899, both men and women from the middle-parental SES group had the shortest average moving distance (Figure 2 and Table S6). In this period, women from the low-SES group moved the farthest, and each SES group differed from one another (Table S6). And for men, only the middle-SES group differed from the other groups in terms of moving distance, i.e., low- and high-SES individuals moved similar distances (Table S6 and Figure 2). In 1900–1969, men from the middle-SES group still dispersed shortest distances, while the high-SES group dispersed longest distances (Figure 2). And women from the low- and middle-SES groups dispersed similarly, but women from the high-SES group dispersed farther than women from the other groups (Figure 2 and Table S6).

Third, the average dispersal distance of all parental SES groups increased from the 1810–1899 time period to the 1900–1969 period for both men and women (Figure 2 and Table S7). Women from the low-parental SES group had the smallest increase in distance dispersed through time, and high- and middle-SES groups had similar increases (Figure 2). However, representatives of low-SES group women were still found to have an increase in the average predicted distance, from 42.2 km (SE = ±1.61 km) to 53.6 km (SE = ±2.20 km; Table S8.). Conversely, for men, the low- and middle-SES groups had a similar increase in time, and the high-SES group demonstrated even more intense growth in dispersal distances (Figure 2), from 41.7 km (SE = ±1.45 km) to 73.3 km (SE = ± 3.17 km; Table S8). All pairwise comparisons of changes in dispersal distance for men and women between the time periods can be found in Table S7, and all predicted distances for each variable grouping can be found in Table S8.

## Discussion

We assessed how parental SES affected dispersal probabilities and dispersal distances of young men and women, as well as how these relations changed over time in Finland between 1760 and 1969. This study utilized accurate individual-level data collected from church records that originated from parishes of four major regions of the country. Overall, we found that the dispersal behavior (probability of dispersal, as well as moving distance) increased over time for both men and women. We also found that dispersal is more likely when young individuals do not have plentiful early life resources and that women disperse more often, but shorter distances, than men. The intensity of changes in dispersal probabilities and distances varied between the parental SES groups. Our results of dispersal behavior at young age are in line with those of similar studies on 19^th^ century Europe (e.g., studies by Baise and Voland^7^ and Kok and Bras^34^). Using a genealogical dataset allowed us to compare individuals across time and space in Finland. Generally, approaches that trace lineages from the present back to the history (ascendants) may lose or underrepresent some childless relatives in the lineage, and descendant data—which trace individuals forward in time across multiple generations—may suffer from missing information, potentially introducing bias.^61^ In contrast to most descendant datasets, which are often limited in size and restricted to small areas or parishes, our dataset is large, spans multiple areas, and parishes across Finland and is unlikely to have underrepresentation of migrants with no children, as all descendants have been followed from birth parish registers (to their destination registers) regardless of whether they had children or not. Therefore, our detailed and comprehensive data from Finland’s church register enable us to reveal more nuances about dispersal behavior from the historical perspective and timely changes better than previous studies.

Long-distance, out-of-parish migrations were not common in pre-industrial European societies, but shorter, within-parish migrations were more frequent^62^^,^^63^; however, after industrialization, all types of migrations increased greatly in Europe.^64^ Accordingly, we found that the overall dispersal probability and dispersal distance increased through time in Finland as we hypothesized, most likely because the environment (society, infrastructure, and culture) had vastly changed in the observed 200 years.^47^^,^^57^^,^^60^ These findings were expected as our first hypothesis was that before industrialization, moving was more difficult and costlier (e.g., less roads and railways, and laws against free moving); job opportunities were limited and shifting from one job to another was difficult; and the social classes had large differences.^57^^,^^59^^,^^65^ After industrialization, dispersal behavior changed in multiple fronts in Finland; opportunities in industrial and urban-related jobs increased; women gained more rights and, therefore, had more possibilities in the labor market; and pursuing education became more accessible for everyone,^59^ i.e., the environment became more favorable for dispersal. However, we found that the dispersal probability and distance changed slightly differently for each parental SES group through time.

Depending on the environment, available parental resources in early adulthood can either help with necessary costs of dispersal or provide opportunity for staying near parents (e.g., Kok^30^ and Aassve et al.^33^). In line with our second hypothesis, we found that the association between parental resources (SES) and dispersal probability varied between the time periods. Individuals from both high- and middle-SES families exhibited an increase in their dispersal probability over time in our sample. Individuals from the low-SES group, on the other hand, had no change in their dispersal probabilities over time. However, we are cautious to draw conclusions about the changes between the 1760–1809 and 1810–1899 time periods due to a quite small sample size in the first mentioned period. Additionally, the low-SES group had a higher probability of dispersal than the representatives of the other groups over all three observation periods, even though they had fewer resources. Therefore, young individuals without substantial family wealth most likely had the highest pressure of acquiring work anywhere they could find it,^30^ and the expected resource benefits from work might exceed the costs of moving, as the neoclassical economic model theorizes.^13^^,^^14^

Moving away from home could have helped low-SES individuals to secure resources for themselves (and even possibly for parents), but it could have caused them to forgo the benefits of living near family, e.g., help from parent with childcare,^66^ and the familiarity with the natal environment.^21^^,^^22^ Even the push and pull theory claims that poverty is one of the pushing factors that influence migration.^12^ Perhaps, individuals from low-SES families are more prone to the unfavorable conditions of the home parish that are pushing individuals away to new location, even though they would have to deal with the costs of leaving their familiar birth parish. For example, in a Chinese population with multiple kinship systems and residence patterns, especially women who had migrated away from natal location for marriage, had increased workload and decreased kin relations compared with those who stayed in their own birth places.^67^ This could indicate that, similar to our case in historical Finland, dispersal was avoided when possible due to its high costs and occurred mostly when there were no other choices. It might explain why individuals from higher-SES families were better able to stay near their birth places to use these kin benefits and parental resources. On the other hand, Towner (1999)^29^ hypothesized that individuals from poorer families had lower dispersal probabilities than middle-class individuals because they could not afford moving away due to high costs of dispersal. However, this was not the case in our study, and the predicted dispersal probabilities of the low-SES group were, on average, 71% in women and 63% in men in the 1810–1899 period. Alternatively, individuals from high-SES families had fairly low predicted dispersal probabilities before industrialization; for example, overall, only 35% of women and 23% of men from high-SES families were predicted to disperse during the 1810–1899 period. This finding strengthens the conclusion that in pre-industrial Finland, philopatry was the desired choice when resources were available from a young age.^22^^,^^24^

We also found that the high-SES group had the greatest change in their dispersal probability over time, with men and women having almost 10 times higher odds of dispersal in the 1900–1969 period compared with the first period (1760–1809). Additionally, in the pre-industrial period (1810–1899), men born to high-SES families dispersed as far away as the low-SES individuals, whereas women from low-SES families moved farther than the high- and middle-SES women. This could be explained by motives for dispersal.^68^ As we discussed earlier, philopatry might have been the more beneficial choice in pre-industrial times. Therefore, perhaps, when dispersing, women ought to move as close to their home parish as possible in order to gain some of the benefits what kin could provide, even if they had to leave their birth parish, which might have been easier for women from middle- and high-SES families. In contrast, dispersing men from high-SES families might have had the knowledge and resources to acquire work farther away as was hypothesized in another study.^9^ In comparison, men from poorer backgrounds might have had less control over where they could move. Our results, therefore, suggest that the representatives of the high-SES group had fewer reasons for leaving their birth parish, but when they did, resources might have helped with the costs of long-distance migrations.

After industrialization, dispersal might have become a better option for children of the wealthier families too. Advances in education or structure of working cultures, i.e., jobs that came with industrialization and urbanization provided opportunities for a lot of people to move to cities.^60^ For example, improved education for all income classes and sexes could have changed the premise of dispersal for everyone. However, pursuing education was more likely the choice for children of wealthier and more educated parents,^39^ as education and employment have been found to be common reasons for long-distance migrations, and people with high income tend to move more often and longer distances because of employment.^68^ Perhaps, after industrialization, the benefits from philopatry were no longer the “optimal” choice, and dispersing provided better opportunities than staying for everyone. In our results, the dispersal probability of individuals from the middle-SES group was between the other two groups through time; however, they more closely resembled the low-SES group than the high-SES group. Therefore, individuals from the middle-SES group should reflect both of these groups; some might have had the need to move for work, and some were able to stay due to their parents’ resources. Individuals from middle-SES families also dispersed the shortest distances, which could indicate having a possibility or wanting to stay near family.

As high-SES individuals showed an increase in their dispersal probability toward the modern period, the gap in the dispersal probability between individuals from higher- and lower-SES families narrowed over time. Additionally, in the later time period of our study, all SES groups exhibited increases in their average dispersal distances, and both men and women in the high-SES group dispersed farther than those in the other groups. The changes in infrastructure such as railways and road networks most likely enlarged the distance that individuals were able to travel. Here, the effect of resources most likely came in handy because traveling to urban areas or traveling for educational purposes required financial support, which could be received from parents.^39^ The large-scale shifts in Finnish society after industrialization are reflected in our results about changes in internal migration over time: high- and middle-SES individuals were affected more by the environmental changes and showed increase in their dispersal probability and moving distance, but among low-SES individuals, dispersal might have been a compulsory phase in life and, that is why, they had the smallest change in dispersal over time.

Some of our results could be explained by the differential inheritance patterns and access to resources of men and women in different time periods.^35^^,^^36^ As our third hypothesis suggested, women were found to have higher dispersal probabilities than men, which is a common trend existing in patriarchal societies.^5^^,^^7^^,^^26^ Interestingly, men and women from low-SES families had the smallest difference between their dispersal behavior, whereas sex differences were the largest among high-SES men and women. One of the main reasons for the smaller sex differences in low-SES families might be that men and women with fewer familial resources are under the same pressure to secure work and living for themselves, which might include moving away from their family. In turn, high familial wealth might affect the dispersal decision more for men than for women; men from high-SES families were most likely in good position for inheritance or for gaining other resources near or with their family, which encouraged them to stay near their birth place (e.g., Pooley and Turnbull,^9^ and Nitsch et al.^28^), whereas women from high-SES families might not have had similar opportunities for inheritance as their male counterparts, even though women in Finland had better opportunities for at least some inheritance from parents, and sometimes first-born women could bring her husband to family home and that way inherit parental home.^35^

In our population, children of farmers, land owners, and crofters belonged to the middle- or high-SES groups, which were more likely to stay than individuals from the low-SES group, suggesting that inheritance practices can affect the dispersal probability. Similarly, previous studies have found that assets and land tied, especially the eldest sons, to their natal sites.^7^^,^^34^ Oldest sons of land owners were usually first in line to inherit family farms and assets, and, therefore, dispersing away from family would not be beneficial or possible for them. Sometimes, parental wealth would be distributed to multiple sons or even daughters. Daughters might have inherited wealth in ways other than land, which did not tie them to their family farm.^35^ Nitsch et al. (2016)^28^ previously studied the effect of siblings, birth order, and SES on dispersal away from natal community in the Finnish population, finding that sons inheriting land owning from parents were more likely to stay than other sons of the same families, but birth order did not affect dispersal in landless families. Our results suggest that women from high-SES families were more likely to stay in their birth parish than women from low-SES families. Women from higher-SES families might have had better chances of marrying within their birth parish than women from poorer families who might have had to move to another parish to marry. Therefore, perhaps, rather than staying due to inheritance, women from high-SES families might have benefitted from staying in their birth parishes and being near their families in other ways as well (location-specific capital, cooperation with kin, familiarity with the environment, etc.).

Another common reason for the detected female-biased dispersal is that women usually moved to their spouse’s home for marriage, and they still are more likely to move, even in the modern society,^69^ whereas men could have moved more in search for work and resources so that they would be able to provide for their family. Although there are sex differences in dispersal behavior across all SES groups, both sexes from low-SES families might have had the highest pressure to leave their birth parishes. In general, men moved longer distances than women in the SES groups and through time, but in both men and women, dispersal distances increased with time. The increased moving distance across time for women could be explained by their changing role in the society, which has been discussed earlier. In addition to traditional marriage-related moves to nearby parishes, women might have started to move elsewhere for education and work-related reasons more often. It is, however, noteworthy to emphasize that many factors contribute to the observed sex differences in dispersal. As women’s rights improved and other major societal changes advanced over time, the sex-specific motives for dispersal could have changed as well. For example, instead of moving for marriage, female migration could be prompted by new educational and professional opportunities that were not available for them previously.^70^ Lento (1951)^59^ noted that the share of women in Finland increased in all economic sectors at the end of 1800s and early 1900s, and, therefore, increasing dispersal in search for jobs became more common for women as well. And still, when a study looked at couples’ moving distances in a modern setting, women were found to move farther away and more often to their spouse than men did.^69^ Women were also more likely to move to a man’s home at the start of their co-residence, indicating that even after major societal changes, women were still more influenced by affiliation when moving. In this light, the consistent sex differences in dispersal shown by our study are not surprising.

We tracked changes in dispersal from 1760s to 1960s, presenting results for the longest period of observation to date. This observation window was rich for societal transitions that created interesting environments for internal migration and finding empirical differences between our three time points, once again highlighting the importance of context in shaping human behavior. We provide new insights into studies of long-term changes in migration patterns in Finland, a country known for late urbanization and industrialization compared with other European countries. Finding similarities in the changes in dispersal after urbanization and industrialization between Finland and other countries stresses the relevance of previous theoretical explanations of historical trends in internal migration. We also explored the interaction between sex and family resources in the changes in dispersal over time, which allowed us getting a more nuanced picture for both the probability and the distance of moves. Focusing on parental SES as a factor for dispersal enabled us to contribute to the discussion on the importance of accounting for the “linked lives” principle—in case of our study, the role of parents—in explaining human behavior over their life courses. Although, over the last decades, there has been a lot of studies about the timing of leaving the parental home, very little is known about the moving distance of young adults when they transition to independence. Therefore, our study provides new insights into the individual characteristics and contexts that are associated with both the dispersal probability and the tendency to move close or far from birth place during young adulthood—a life stage that is characterized by transition to adulthood but still a high dependence on parental resources—through times and societies.

### Limitations of the study

Our analytical division of the time periods is coarse, and future research could employ a cohort perspective to the analyses in order to draw a more detailed picture of changes in dispersal behavior over our uniquely long period of observation. We explored dispersal only on the individual level without focusing on families; some of the moves in our data could have been undertaken by whole families. Future research could focus on examining whether the representatives of some families were more likely to leave the birth place, which characteristics of the family beyond the SES were associated with dispersal of multiple family members, whether the same familial characteristics were important over time, and how death of a parent influenced dispersal for different SES groups. Future studies could also focus on the own SES of individuals and compare it with parental SES for those who left the birth place and those who did not. Also, we could have utilized the socioeconomic classification tool called Historical International Standard Classification of Occupations (HISCO)^71^ to classify our SES variable; doing so could have facilitated harmonization with data from other countries and enabled international comparisons. However, historically, Finland was a poor agrarian society with very few educated or schooled people. Although these skilled and educated individuals had a high status in society, social status in historical Finland was mostly defined by land ownership. Therefore, our classification better describes the socioeconomic differences in our study system. Some of our study’s limitations stem from the absence of data on the motives of leaving the birth place. It would be valuable to analyze how the reasons for moving changed for men and women and for the representatives of SES groups; in this study, we could only speculate about the motivation.

It is worth considering that in Western European countries, life cycle service (on farms or as domestic servants in urban places) was commonly performed by young, single people, suggesting that there were many circular migrations during youth in the past,^30^^,^^34^ while, during the industrialization period, these trends changed.^72^ Looking at the sequence of moves rather than focusing on first moves from the birth parishes as was done in our research would enrich our understanding of societal transformation of Finland from 1760 to 1969 and should be explored in future studies. Our study is based on Finnish data, but our hypotheses and interpretations were formulated using the models of internal migration and results of previous research from various countries. Although the results are generally in line with those of previous studies from Europe and North America, the explanations for them are not always clear, and the results are not easily generalizable to other contexts. There is a growing number of digitalized individual-level historical records that should be harmonized to test the universality of theoretical models explaining internal migration, including dispersal, and making formal international comparisons. Constructing and using such data would enable researchers to delve deeply into the structural differences between Finland and other societies and enhance the theoretical depth of the discussion.

Another limitation of our study is that we did not consider the environmental factors for migration, which can relate to the economic, political, social, and demographic contexts or affect migration decisions directly. The environment drives migration through mechanisms characterized as the availability and reliability of ecosystem services and exposure to hazard.^73^ One example of environmental factors is climate change. As time goes by, the trend of population migration caused by climate change is expected to intensify. However, there is no evidence that there is within-country or even out-of-country migration in Finland due to climate change.^74^ Importantly, although we speculate about the major role of industrialization and urbanization in changes in dispersal behavior, we do not rule out the interference of other historical events (e.g., Finland’s independence and World War 2). We admit that multiple factors likely acted together. Future studies should focus on estimating the effect of key events in the history of Finland on dispersal.

Last, we calculated the dispersal distance based on the coordinates of the parish church, which theoretically might overlook the differences in actual residence. However, according to Niedomysl et al.^75^ who compared the actual distances with the distances between different regional centroids, the distances inferred from municipal area centroids are acceptably accurate estimates. Because parish areas are very similar to municipalities, our proxy can be considered reliable.

## Resource availability

### Lead contact

• Requests for further information and resources should be directed to and will be fulfilled by the lead contact, Jenni Kauppi (jenni.j.kauppi@utu.fi).

### Materials availability

• This study did not generate new unique reagents.

### Data and code availability

• Pseudonymized data reported in this paper will be shared by the lead contact upon request.
• Any additional information required to reanalyze the data reported in this paper is available from the lead contact upon request.
• All original code is available in this paper’s supplemental information.

## Acknowledgments

The authors acknowledge funding from the 10.13039/501100005781Kone Foundation (V.L. and J.J.K., grant number 202108374; “MigrantLives”), Strategic Research Council (10.13039/501100009047SRC) within the Research Council of Finland to NetResilience consortium (V.L., M.L., and M.S., grant number 364385; and A.A., 364382 and 364371; “NetResilience”), the European Research Council to KinSocieties (V.L., ERC-2022-ADG, grant number 101098266), Profi7 program by Research Council of Finland to Human Diversity consortium (V.L. and M.L., grant number 352727), the Centre of Excellence (V.L., grant number 374221) by Research Council of Finland, and from the 10.13039/501100002341Research Council of Finland (M.L., grant number 371390).

## Author contributions

Conceptualization and study design, J.J.K., M.S., M.L., A.A., and V.L.; data curation, J.J.K., M.S., and M.L.; formal analysis, J.J.K.; methodology, J.J.K. and M.S.; writing – original draft, J.K. and A.A.; writing – review & editing, J.J.K., A.A., M.S, M.L., and V.L.; project administration and supervision, V.L.

## Declaration of interests

The authors declare no competing interests.

## STAR★Methods

### Key resources table

**Table:** 

REAGENT or RESOURCE	SOURCE	IDENTIFIER
**Deposited data**		

Data used in this study	Available through the lead contact	Jenni Kauppi jenni.j.kauppi@utu.fi

**Software and algorithms**		

R version 4.4.2	R Core Team^76^	https://cran.r-project.org/
Code used in this study	Supplementary material	Supplementary material: R code

### Experimental model and study participant details

Omitted as our study does not involve biological models.

### Method details

#### The Finnish population

The 18^th^ and 19^th^ century Finland was a so-called estate society in which individuals were classified into estates and social status was inherited from parents. Belonging to an estate determined individual’s social position in the society, and the wealthiest had special rights compared to others. Majority of the population (70%) were outside the special rights and had no constitutional rights of any kind; in Finland the estates were nobility, clergy, bourgeoisie and peasantry. An example of groups outside the estates are such as landless peasants, farmhands and other servants, and “parasites” who lived in miserable conditions and often worked for food (examples of low SES group). The wealthiest (examples of high SES group) of the population were the nobility – only a few percent of the population – who lived in manors with servants, the clergy who were educated and literate, and landowners who owned and farmed their own lands. Others belong to somewhere in between these two (however not everyone had rights); peasants (small or mediate landowners) those who farmed smaller lands and had some rights (compared to crofters) owned their lands and farms, were self-sufficient and got their income from those lands, few were bourgeoisie in the cities and had rights for trading, also tenant farmers (landless who rented farming lands), artisans and workers (examples of middle SES group). However, multiple changes were happening at the end of the 1800s which resulted in transformation of the society.^58^

In the late 19^th^ century, the industrialization in Finland was in progress and caused social and economic changes in the country, especially the Second Industrial Revolution at the end of 1800s. Additionally, there was national awakening (“kansallinen herääminen” *in Finnish*) in 1860s and 1870s when the economy prospered, forest industry developed greatly and transport connections improved.^59^^,^^65^ In the end of 19^th^ century the society transformed from an estate society, in which individual’s rights were determined by their estate, to a class society in which instead of inheriting status from parents, earning wealth became more important. However, Finland was an agrarian society for long after industrialization had begun; in 1918 70% of the population was working in agriculture and still 57% by 1940.^60^ Finland then slowly changed into a civil society toward the 1900s when more schools and libraries were built, a Finnish speaking civil-society developed, and more Finnish newspapers were created.^65^ The laws which limited the moving between parishes were also lifted at the end of the 1800s, which opened more possibilities for dispersal. At the same time, the inequality between wealthy and poor had increased and income differences were exceptionally high.^58^ Additionally, other specific events happened at different time periods, such as legal changes in women’s position (right for equal inheritance in 1878, voting rights in 1917), the famine in the years 1867-68, gaining independence in 1917, and different wars in each time period e.g., civil war and the Second World war, had a substantial influence on the Finnish society. All these events created different environments for people and could transform dispersal behavior over time. It remains unknown how availability of resources functioned for men and women making their locational choices in different periods of time in Finland.

#### Church books

Finland has comprehensive long-term demographic information about its population, because the Lutheran Church had collected data from all individuals in their parishes into church registers, which has been legally required since 1749 onwards in the whole country.^56^ The dataset (*n* = 98,135) used in this study has been collected from these church records by a team of professional genealogists who constructed it from approximately 700 randomly selected founding mothers and 700 founding fathers and their lineages across time. The family lineages of this study originate from eight parishes in four major regions in the 18^th^ century: Southwest Finland: Hiittinen, Rymättylä, and Kustavi; Pirkanmaa region: Ikaalinen and Tyrvää; Northern Ostrobothnia: Pulkkila; and Karelian parts: Jaakkima and Rautu. These data contain information such as individuals’ birthdays, all marriage information (dates and spouses), all children, date of death, and records of all movements (see e.g.,^28^^,^^70^^,^^77^^,^^78^). In Finland, each parish maintained records of incoming and outgoing individual movements (“Book of Migrations”) which include information on date of move and its destination and origin parish. Therefore, we were able to follow our focal individuals from birth parish records to the destination records and record all of their life events regardless of location. Some individuals may have been censored due to missing parish records for certain locations or years, migration out of the study area (e.g., to another country), or unavailability of records at the time of data collection. These cases have been included in our analyses as censored individuals using their last date of known appearance.

### Quantification and statistical analysis

To test our hypotheses concerning dispersal, we utilized a sample of 22,429 individuals for whom we had information on sex (men and women, deducted from names in the church records), parental socioeconomic status (high, middle, and low), birth region (4 different areas), dispersal parish, and time of birth and dispersal (in the years between 1760 and 1960; STAR Table A). The dispersal variable was determined as here as leaving the birth parish and coded as binomial variable between the age of 15–35 (dispersal = 1) or staying in the birth parish (dispersal = 0) if there was no record of moving or the recorded move occurred within birth parish. This was done by comparing the birth parish name to the name of the dispersal parish recorded, i.e., if the parish names were different from each other dispersal was classified as “1”. Those who had no written record of dispersal parish must have had their life history followed at least to the age of 35 years to be included in the sample, to ensure the missing record of dispersal parish was not in fact missing data but rather that there was no record because the person did not leave their birth parish. We were interested in early adulthood dispersal, and most dispersal away from birth parish happened before the age of 35 (Figure S1), explaining the cut-off ages for dispersal. We only had records of out of parish movements not out of home moves, therefore individuals might have moved away from their families within the parish but this did not count to us as dispersal is this study setting. We also excluded all dispersal information of Karelians that were evacuated during World War II, during the years between 1939 and 1945 (n excluded = 1631), to exclude any effects that the forced migration might have on our analyses. A flowchart of this study (Figure S2) illustrates the final sample size and the number of observations excluded due to missing values in any of these variables.

**STAR Table A:** The sample sizes of each time period and parent’s SES of men and women (n = 22,429). Percentages are for dispersal (no/yes) for each SES group by sex.

		Women		Men	
		Dispersal n (%)			
Time period	Parental SES	no	yes	no	yes
1760–1809	Low	11 (32.4%)	23 (67.6%)	6 (33.3%)	12 (66.7%)
	Middle	40 (62.5%)	24 (37.5%)	34 (66.7%)	17 (33.3%)
	High	295 (79.3%)	77 (20.7%)	329 (83.9%)	63 (16.1%)
1810–1899	Low	370 (34.8%)	694 (65.2%)	399 (41.3%)	566 (58.7%)
	Middle	1133 (44.9%)	1393 (55.1%)	1245 (51.9%)	1155 (48.1%)
	High	1852 (58.8%)	1299 (41.2%)	2068 (68.0%)	972 (32.0%)
1900–1969	Low	368 (37.5%)	613 (62.5%)	410 (44.4%)	514 (55.6%)
	Middle	852 (47.6%)	937 (52.4%)	961 (54.8%)	792 (45.2%)
	High	669 (47.0%)	754 (53.0%)	830 (56.0%)	652 (44.0%)

The parental socioeconomic status (SES group, low middle, high) for each individual was determined by their father’s socioeconomic status —if an individual’s father’s SES was missing, we used the mother’s SES— when the parent was around 30 years old, which reflected their wealth and educational status in the society. The father’s SES was used in 95% of cases, as during the period covered by our study the father’s occupation and social status were typically the primary determinants of the family’s socioeconomic position in the society. The parental SES included three categories: high, middle, and low. These categories were manually coded based on the parents’ occupation and educational level reflecting achieved status in the society at the time. The high SES group includes wealthy (e.g., landowners) and educated individuals (e.g., clergy, few nobility), the middle SES group includes individuals with the average income (e.g., tenant farmers, craftsmen, small-land farmers), and the low SES are represented by the poor and less educated (e.g., servants, dependent lodgers). The categorization was performed to account for possible comparison of occupations and statuses between times; as the society changed, the sorting changed as well. Thus, the historical part (before 1850) reflects mostly landownership, while the modern part (after 1850) reflects education and labor workers (see Salonen et al. (2024)^79^ who has used this categorization before). We utilized the SES of an individual’s parent when the parent was 30 years old, instead of the main persons’ own status, as the explanatory variable because we were interested in the role of resources available to individuals when leaving the birthplace. The three-level categorization captures important differences between the groups that might influence lifetime successes in survival and reproduction.

Mother’s unique identification code (ID) was used to take into account potential family clustering of individuals (siblings from same family). Considering that in Finland, similar to other countries, regional development has progressed unevenly,^80^ we took into account potential regional differences in dispersal. The regions were divided into four larger groups based on birth region: Northern (includes: Kainuu, Central Ostrobothnia, Lapland, Ostrobothnia, North Ostrobothnia), Central (includes: Eastern Uusimaa, Kanta-Häme, Central Finland, Pirkanmaa, Päijät-Häme, Uusimaa), Southwest (includes: Åland Islands, Satakunta, Southwest Finland) and Eastern Finland (includes: South Karelia, South Savo, Ingria, Karelia, Kymenlaakso, North Karelia, North Savo, North Russia).

Three time periods were selected due to their clear societal and environmental characteristics during the 18^th^ and the 20^th^ centuries in Finland. The first period (from 1760 to 1809) represents the time before The Finnish War, which was fought between the Kingdom of Sweden (of which Finland was part of) and the Russian Empire. The war ended in 1809 and Finland became an autonomous Grand Duchy of Finland within the Russian Empire (the second period: from 1810 to 1899). The third period (from 1900 to 1969) starts at the beginning of the 1900s because at the end of the 1800s industrialization and transition to a class society had begun, and in 1917 Finland gained independence from Russia. We assigned individuals into three time periods based on their dispersal year. For example, if an individual dispersed in 1850, they were assigned to the 1810–1899 period. We calculated a comparable year for those who did not disperse to categorize them into the three time periods, i.e., for those who stayed in their natal parish, the calculations were based on the average age of dispersal, which was 23 years for women and 25 years old for men. Therefore, the time period for individuals who did not move was determined based on the year they were 23 years (women) or 25 years (men) old.

All analyses were carried out with R version 4.1.3.^76^ In our analyses, we used a generalized linear mixed-effects model (GLMM, ‘*glmer*’ from *lme4* package;^81^) to explored the factors associated with dispersal probability which was coded as a binary variable (1 = leaving, 0 = staying). The predictors used include sex, parental SES, time period, as well as an interaction between parental SES and time period (to test if dispersal probability changes differently with time in different parental SES groups), parental SES and sex (to test if men and women have different dispersal probabilities depending on their parental SES), and sex and time period (to investigate if possible difference of men and women’s dispersal probability changes in time). Three-way interaction between the variables was removed from the final model, because, according to the likelihood ratio test (‘*lrtest*’ from *lmtest* package^82^), there was no statistically significant difference between reduced model and full model (LogLik = −13764, Chi-square: 1.975, *p*-value 0.740). The model accounts for potential regional variation and familial clustering by incorporating random variables of region and mother’s ID. We used the binomial family with a logit link function to accommodate the binary nature of the outcome. The optimization process (using ‘*allFit*’ function from *lme4* package selecting the best optimizer i.e., highest log likelihood and no convergence warnings) resulting in ‘*bobyqa*’ optimizer within the ‘*glmerControl*’ framework. We examined our predictors for multicollinearity using generalized variance inflation factors (GVIF) which was done with ‘*vif*’ *function from the package car*^83^*.* All adjusted GVIF^1/(2∗Df)^ values were below the conventional thresholds, indicating no problematic collinearity. Also, residual diagnostics were evaluated using *DHARMa*^84^ package, with ‘*simulateResiduals*’ function where the QQ plots show approximate adherence to the expected 1:1 line. A minor overdispersion of value 1.023 indicate negligible overdispersion. To assess the significance of the fixed effects in our model, we utilized the ‘*Anova*’ function from the package *car.*^85^ Specifically, we used the Type III Wald χ^2^ Test to evaluate the importance of each predictor variable while accounting for the random effects structure introduced by regional and family variations. Comparisons and estimated marginal means were computed using the *emmeans* package^86^ to examine pairwise differences between levels of categorical predictors. All plots were created with *ggplot2* package.^87^

To further investigate dispersal behavior, we focused on the moving distances for those who had moved away from their birth parish (*n* = 8439, dispersal = 1, STAR Table B). In these analyses, we only included the time periods 1810–1899 and 1900–1969, because of the small sample size of dispersing individuals in 1760–1809, impacting the reliability of analyses. The distance from the birth parish to the dispersal parish was computed using the coordinates of the parish’s church, i.e., from coordinates of birth parish church to coordinates of dispersal parish church. In case where there were multiple churches, the coordinates of the main church were used because most villagers resided close to this site. Finland’s parish names^88^ generally correspond to the names of the municipalities in which they are located and therefore we are able to get the church’s coordinated based on the name of the parish. Out of country moves were removed from this sample, as we could not reliably calculate the distance to other countries, and we were mostly interested in how far individuals dispersed within the country borders. Distances were calculated by using *geosphere* package^89^ in R version 4.1.3.^76^ and computing the great-circle distance of two coordinate points. True distances by road routes were not used because of the absence of accurate coordinates of the individual homes in the mentioned time periods. However, because all distances were created consistently, we could get comparable results. Region was not included as a random effect in this model, as our continuous response variable (dispersal distance) already captures spatial variation. Including region led to convergence issues due to collinearity with dispersal distance. The model accounts for potential familial clustering by incorporating random variable of mother’s ID. A flowchart of this study (Figure S2). shows the final sample size and the number of excluded observations based on missing values on any of these variables.

**STAR Table B:** The average dispersal distance for men and women through time and by SES (n = 8439). The sample sizes, the average moving distances, the standard errors, and the shortest and the longest distances are given for men and women separately in each category

	Time period	SES	Sample size	Average distance moved (km)	Standard error	Range of distance moved (km)
Women	1810–1899	Low	620	68	3.4	2.5–545
		Middle	1180	48	1.7	2.5–603
		High	1085	54	1.9	6.5–506
	1900–1969	Low	539	78	3.7	6.5–720
		Middle	817	85	3.1	2.5–720
		High	661	100	4.3	6.5–766
Men	1810–1899	Low	470	66	3.4	2.5–507
		Middle	941	55	2.2	5.5–540
		High	711	63	2.7	6.5–505
	1900–1969	Low	398	106	5.7	1.4–683
		Middle	545	99	4.6	1.4–918
		High	472	123	5.5	6.5–586

We used a linear mixed-effect model (LMM, R *lme4* package*,* with *‘lmer*’ function^81^*)* to test the hypotheses related to dispersal distances. In the model we included as explanatory variables sex, time period, parental SES, all possible two-way interactions and sex ∗ time period ∗ parental SES-interaction (to test if dispersal distances of men and women change with time in different parental SES groups), and the random term of mother’s ID (to account for familial variation). The response variable was the distance between birth parish and dispersal parish which was transformed into logarithmic values because of a right skew of our distance data. The logarithmic values followed a normal distribution. The optimization process (using ‘*allFit*’ function from *lme4* package selecting the best optimizer i.e., highest log likelihood and no convergence warnings) resulting in the default *“nloptwrap”* optimizer within the ‘*lmerControl*’ framework. We examined our predictors for multicollinearity using generalized variance inflation factors (GVIF) which was done with ‘*vif*’ *function* from the package *car*^83^*.* All adjusted GVIF^1/(2∗Df)^ values were below the conventional thresholds, indicating no problematic collinearity. Also, normality of residuals war checked by examining residual plots and QQ plots which indicated normality and homoscedasticity. Estimates of differences in dispersal distance between the SES, time and sex, were computed with *emmeans* package*,*^86^ using pairwise comparisons of different groups. To assess the significance of the fixed effects in our model, we utilized the ‘*Anova*’ function from the *car* package.^85^ Specifically, we used the Type III Wald χ^2^ Test to evaluate the importance of each predictor variable while accounting for the random effect.
