# Supplementary material for: Socioeconomic status influenced dispersal in early adulthood in Finland from 1760 to 1969
Source: iScience. 2026 Mar 25;29(4):115467. doi: 10.1016/j.isci.2026.115467 (PMC13091024; doi:10.1016/j.isci.2026.115467)
Supplement: Document S1. Figures S1–S2, Tables S1–S8, and Data S1 [file mmc1.pdf]

iScience, Volume *29*

Supplemental information

Socioeconomic status influenced dispersal in early adulthood in Finland from 1760 to 1969

Jenni J. Kauppi, Alyona Artamonova, Milla Salonen, Mirkka Lahdenperä, and Virpi Lummaa

SUPPLEMENTAL INFORMATION

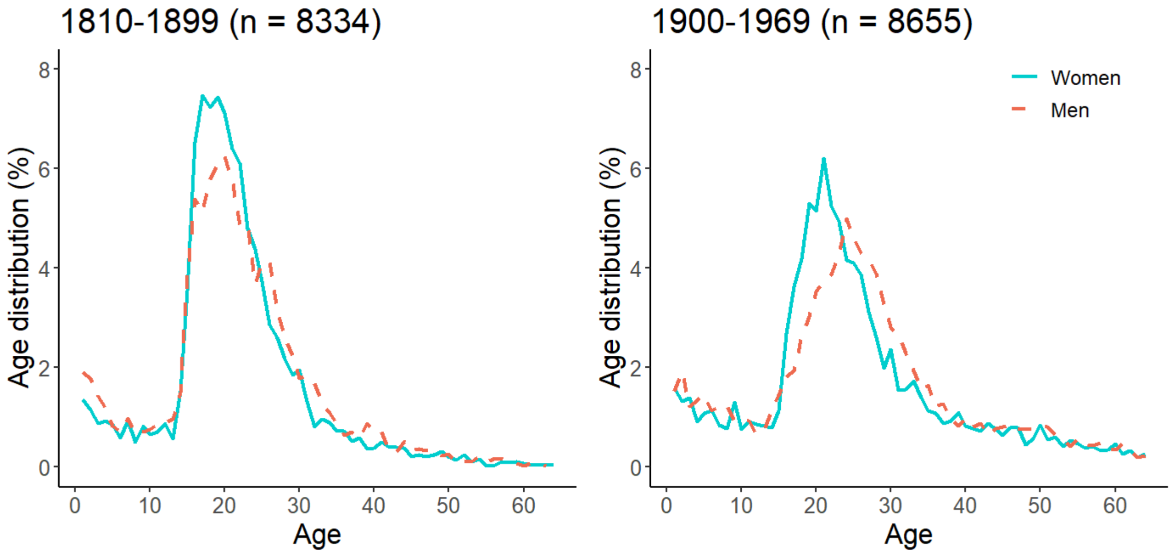

Figure S1. *Distribution of age in percentages of all who dispersed (dispersal = yes) in 1810-1899 and 1900-1969 for men (red, dashed line) and women (blue, solid line).*

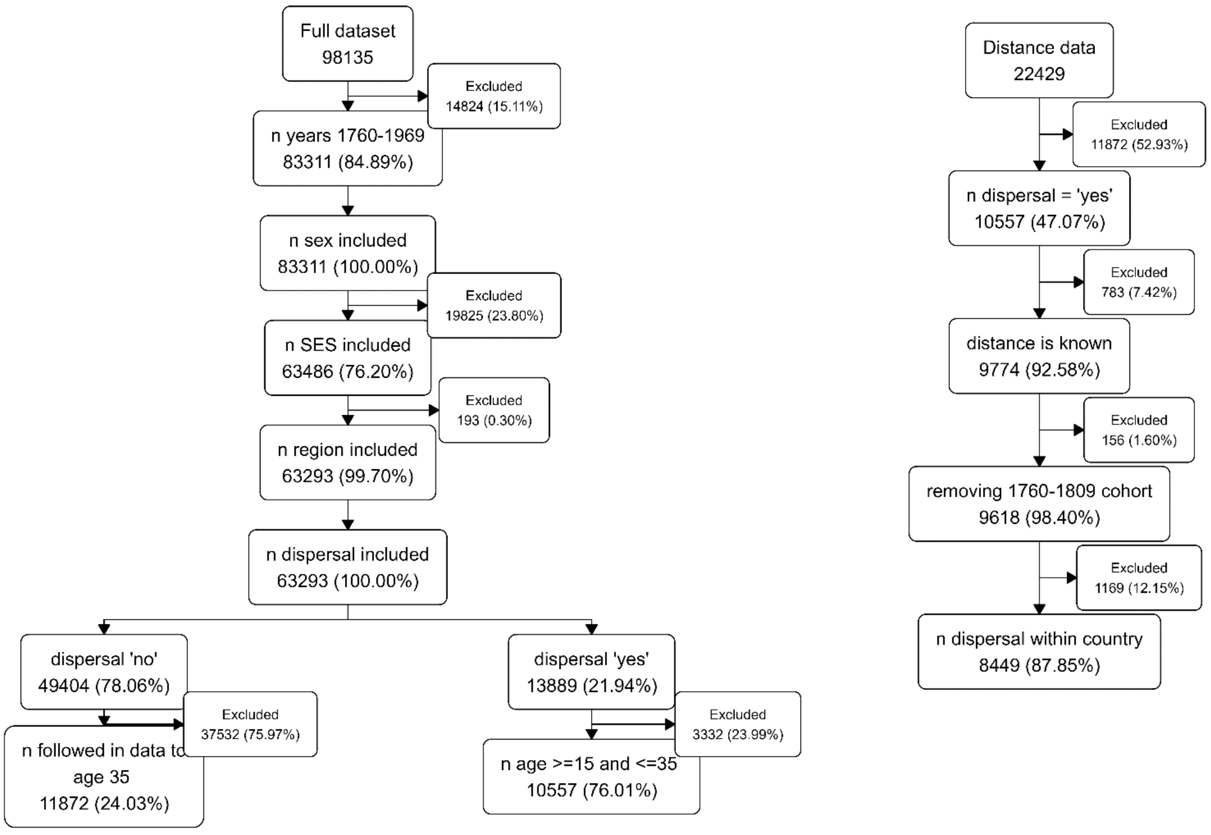

Figure S2. Flowcharts illustrating the sample selections steps. *The flowchart on the left illustrates the included variables in the probability analyses (n = 22429) with excluded missing values for time, sex, SES, region, dispersal (0/1) and age. Additionally, 1631 individuals from Karelia who were evacuated due to WW2 between the years 1939-1945 were excluded from the data. The flowchart on the right shows the subset of available distance data (n= 8449) representing those who dispersed, and the time cohort 1760-1809 was excluded. Percentages of excluded and included cases are calculated relative to the sample size at each preceding step in the flowchart.*

Table S1. *Type III Wald Chi-Square test results from a Generalized Linear Mixed Model (GLMM) showing the effect of predictor variables on dispersal probability (binary outcome), and variance and standard deviation of random effects. Level of significance p <0.05.*

**Effect**		**X^2**	**Df**	**P-value**
**Fixed effect**	(Intercept)	70.42	1	<0.0001
	Sex	8.42	1	0.0037
	Time period	177.22	2	<0.0001
	Parental SES	45.40	2	<0.0001
	Time period * parental SES	88.44	4	<0.0001
	Sex * parental SES	7.48	2	0.0238
	Sex * Time period	0.39	2	0.8231
		**Variance**	**SD**	
**Random effect**	Mother's ID	2.74	1.66	
	Region	0.07	0.26	

Table S2. *The odds ratios of dispersal probability by parental SES groups within the time periods. The second mentioned SES group in comparison is the reference group for odds ratio. SE = standard error. *the lower and upper confidence limits are asymptotic.*

**Parental SES comparison**	**Sex**	**Time period**	**Odds ratio**	**SE**	**Z ratio**	**LCL***	**UCL***	**p-value**
Low vs. Middle	Women	1760-1809	7.895	4.715	3.460	2.449	25.452	0.0005
		1810-1899	1.633	0.184	4.352	1.309	2.036	<.0001
		1900-1969	1.549	0.187	3.619	1.222	1.964	0.0003
	Men	1760-1809	8.549	5.126	3.579	2.640	27.689	0.0003
		1810-1899	1.768	0.204	4.942	1.410	2.217	<.0001
		1900-1969	1.678	0.203	4.274	1.323	2.127	<.0001
Middle vs. High	Women	1760-1809	3.243	1.195	3.193	1.575	6.678	0.0014
		1810-1899	2.792	0.242	11.851	2.356	3.308	<.0001
		1900-1969	1.123	0.121	1.079	0.910	1.386	0.2804
	Men	1760-1809	3.885	1.439	3.665	1.880	8.028	0.0003
		1810-1899	3.344	0.297	13.572	2.809	3.981	<.0001
		1900-1969	1.345	0.144	2.777	1.091	1.658	0.0055
Low vs. High	Women	1760-1809	25.607	13.231	6.276	9.302	70.496	<.0001
		1810-1899	4.558	0.505	13.692	3.668	5.664	<.0001
		1900-1969	1.740	0.219	4.396	1.359	2.227	<.0001
	Men	1760-1809	33.216	17.254	6.744	12.000	91.939	<.0001
		1810-1899	5.912	0.677	15.530	4.725	7.399	<.0001
		1900-1969	2.257	0.284	6.477	1.764	2.887	<.0001

Table S3. *The odds ratios of dispersal probability of time periods by sex and parental SES. The second mentioned SES group in comparison is the reference group for odds ratio. SE = standard error. *the lower and upper confidence limits are asymptotic.*

Time period comparison	Sex	Parental SES	Odds ratio	SE	Z ratio	LCL*	UCL*	p-value
1810-1899 vs. 1760-1809	Women	High	3.547	0.671	6.691	2.448	5.139	<.0001
		Low	0.625	0.315	-0.932	0.233	1.678	0.3512
		Middle	3.044	1.069	3.168	1.529	6.056	0.0016
	Men	High	3.580	0.702	6.506	2.438	5.258	<.0001
		Low	0.631	0.324	-0.897	0.231	1.724	0.3823
		Middle	3.071	1.117	3.086	1.506	6.263	0.0019
1900-1969 vs. 1760-1809	Women	High	9.990	2.017	11.398	6.725	14.841	<.0001
		Low	0.676	0.342	-0.774	0.251	1.821	0.4314
		Middle	3.448	1.224	3.487	1.719	6.913	0.0005
	Men	High	9.579	1.987	10.891	6.379	14.385	<.0001
		Low	0.648	0.334	-0.842	0.236	1.778	0.4080
		Middle	3.306	1.214	3.256	1.609	6.789	0.0010
1900-1969 vs. 1810-1899	Women	High	2.817	0.274	10.647	2.328	3.408	<.0001
		Low	1.081	0.134	0.630	0.848	1.378	0.5625
		Middle	1.133	0.103	1.370	0.948	1.355	0.1733
	Men	High	2.675	0.259	10.151	2.212	3.235	<.0001
		Low	1.027	0.128	0.213	0.804	1.311	0.8605
		Middle	1.076	0.098	0.805	0.900	1.287	0.4151

Table S4. *Type III Wald Chi-Square test results from a Linear Mixed Model (LMM) showing the effect of predictor variables on dispersal distance, and variance and standard deviation of random effect. Level of significance p <0.05.*

Effect		X^2	Df	P-value
**Fixed effect**	(Intercept)	15319.42	1	<.0001
	Sex	6.00	1	0.0143
	Parental SES	28.21	2	<.0001
	Time period	89.01	1	<.0001
	Sex : parental SES	3.36	2	0.1868
	Sex : Time period	3.73	1	0.0534
	SES: Time period	14.19	2	0.0008
	Sex : parental SES : Time period	6.88	2	0.0321
		**Variance**	**SD**	
**Random effect**	Mother's ID	0.3746	0.6121	

Table S5. *Pairwise comparisons of sex differences in dispersal distance through time and by parental SES. Pairwise comparisons of estimated marginal means with log values back-transformed after comparison, contrast indicates the compared variable, ratios are the ratios of the geometric means. SE = standard error. *the lower and upper confidence limits are asymptotic.*

Sex comparison	Time period	Parental SES	Ratio of geometric mean	SE	Z ratio	LCL*	UCL*	p- value
Men vs. Women	1810-1899	Low	0.996	0.049	-0.074	0.905	1.097	0.9413
		Middle	1.107	0.040	2.838	1.032	1.187	0.0045
		High	1.100	0.043	2.449	1.019	1.187	0.0143
	1900-1969	Low	1.201	0.063	3.513	1.084	1.330	0.0004
		Middle	1.066	0.047	1.432	0.977	1.162	0.1522
		High	1.240	0.060	4.455	1.128	1.363	<0.0001

Table S6. *The pairwise comparison of dispersal distance among parental SES groups by sex and time period. The logarithmic values of the distance were back-transformed in the pairwise comparisons (ratio is expected values (means) of the original scale distance). *the lower and upper confidence limits are asymptotic for the ratio of geometric mean.*

Parental SES comparison	Sex	Time period	Ratio of geometric mean	SE	Z-ratio	LCL*	UCL*	p-value
Low / High	Women	1810-1899	1.115	0.054	2.257	1.014	1.225	0.0240
		1900-1969	0.906	0.051	-1.777	0.812	1.010	0.0756
	Men	1810-1899	1.010	0.056	0.173	0.906	1.125	0.8630
		1900-1969	0.877	0.055	-2.082	0.775	0.992	0.0374
Middle / High	Women	1810-1899	0.874	0.036	-3.322	0.807	0.946	0.0009
		1900-1969	0.915	0.047	-1.753	0.828	1.011	0.0796
	Men	1810-1899	0.879	0.041	-2.794	0.803	0.962	0.0052
		1900-1969	0.786	0.046	-4.125	0.701	0.881	<0.0001
Middle / Low	Women	1810-1899	0.784	0.037	-5.146	0.714	0.860	<.0001
		1900-1969	1.010	0.054	0.186	0.910	1.121	0.8527
	Men	1810-1899	0.870	0.046	-2.639	0.785	0.965	0.0083
		1900-1969	0.896	0.054	-1.812	0.796	1.009	0.0699

Table S7. *The pairwise comparisons of change in dispersal distance over time for men and women in each parental SES group. The logarithmic values of the distance were back-transformed in the pairwise comparisons (ratio is expected values (means) of the original scale distance). SE = standard error. *the lower and upper confidence limits are asymptotic for the ratio of geometric mean.*

Time period comparison	sex	Parental SES	Ratio of geometric mean	SE	z-ratio	LCL*	UCL*	p-value
1900-1969 vs. 1810-1899	Women	Low	1.268	0.069	4.375	1.140	1.410	<.0001
		Middle	1.634	0.070	11.387	1.501	1.778	<.0001
		High	1.561	0.074	9.434	1.423	1.712	<.0001
	Men	Low	1.528	0.094	6.887	1.354	1.724	<.0001
		Middle	1.573	0.077	9.316	1.430	1.731	<.0001
		High	1.759	0.097	10.292	1.580	1.959	<.0001

Table S8. *Predicted dispersal distances (km) of men and women in each time period for each parental SES group. SE = standard error. *the lower and upper confidence limits are asymptotic*

Time cohort	Sex	Parental SES	Predicted dispersal distance (km)	SE	LCL*	UCL*
1810-1899	Women	Low	42.2	1.61	39.208	45.514
		Middle	33.1	0.93	31.330	34.990
		High	37.9	1.11	35.781	40.146
	Men	Low	42.1	1.80	38.707	45.768
		Middle	36.6	1.12	34.507	38.902
		High	41.7	1.45	38.950	44.627
1900-1969	Women	Low	53.6	2.20	49.427	58.049
		Middle	54.1	1.84	50.612	57.821
		High	59.1	2.24	54.910	63.713
	Men	Low	64.3	2.96	58.779	70.378
		Middle	57.6	2.26	53.379	62.255
		High	73.3	3.17	67.385	79.829

Data S1: R code, related to STAR Methods.

```
#R script
library("ggplot2")
library(dplyr)
library(lme4)
library(car)
library(emmeans)


#ses data
SESdata <- fulldata %>%
  filter(( (!is.na(age_move) & (deathage >= age_move | is.na(deathage) ) & age_move >= 15 & age_move
< 36) | ( is.na(age_move) & dispersal == "no" & (lastapp - byear) > 34 ))) %>%
  filter(!is.na(SES_parent), !is.na(region_new), !is.na(sex), !is.na(dispersal), BD_year >= 1760,
  BD_year < 1970 ) %>%
  filter( !(region == "Karjala" & moveyear %in% c(1939:1949) ) )


#ses model
modelSES<- glmer(dispersalbinom ~ sex + centuryCohort + SES_parent  +
                    centuryCohort*SES_parent + sex*SES_parent + sex*centuryCohort +
                    (1 | mumid) + (1 | region_new),
                  data = SESdata
                  , family = binomial(link = "logit")
                  , control = glmerControl(optimizer = 'bobyqa' ))
summary(modelSES)
Anova(modelSES, type= 3)
#pairs and emmeans
pairs(emmeans(modelSES, ~ SES_parent*centuryCohort, by = c("sex", "centuryCohort") ,
              type = "response"),
      comparison = TRUE,
      adjust = "none")
confint(pairs(emmeans(modelSES, ~ SES_parent*centuryCohort, by = c("sex", "centuryCohort") ,
                      type = "response"),
              comparison = TRUE,
              adjust = "none"))


ses.emm.scC <- emmeans(modelSES, ~ SES_parent*centuryCohort , by = "sex", type = "response")
SESemmSC <- emmeans(modelSES, ~ SES_parent*centuryCohort , by=c("sex","centuryCohort"), type = "resp
onse")
pairs(emmeans(modelSES,
              ~ SES_parent*centuryCohort, by = c("SES_parent", "sex")  ,
              type = "response"), adjust = "none" )
```

```r
pairs(emmeans(modelSES,
              ~ SES_parent*sex, by = c("centuryCohort")  ,
              type = "response"), adjust = "none" )


pairs(emmeans(modelSES,
              ~ SES_parent*sex,  by = c("SES_parent") ,
              type = "response"), adjust = "none" ) %>% plot()
pairs(emmeans(modelSES,
              ~ SES_parent*centuryCohort, by = c( "sex","centuryCohort")  ,
              type = "response"), adjust = "none" )


pairs(emmeans(modelSES,
              ~ SES_parent*centuryCohort, by = c("SES_parent", "sex")  ,
              type = "response"), adjust = "none" )
ses.pairs.byC<- pairs(emmeans(modelSES, ~ SES_parent*centuryCohort ,
                                   type = "response", by = c("sex", "centuryCohort") ),
                           adjust = "none", reverse = T)
confint(ses.pairs.byC)


ses.pairs.bySC <- pairs(emmeans(modelSES, ~ SES_parent*centuryCohort ,
                                     type = "response", by = c("sex", "SES_parent") ),
                             adjust = "none", reverse = T)



df.SESpairsbyC <-  merge(data.frame(ses.pairs.byC), data.frame(confint(ses.pairs.byC) ) )


df.SESpairsSC <- merge(data.frame(ses.pairs.bySC), data.frame(confint(ses.pairs.bySC) ) )


#plot SES
grid.arrange(
  ggplot(as.data.frame(ses.emm.scC) %>% filter(sex == "M"),
         aes(x = centuryCohort, y = prob,
             group = factor(SES_parent, levels = c("Low","Middle", "High")) ,
             shape = factor(SES_parent, levels = c("Low","Middle", "High")),
             colour = factor(SES_parent, levels = c("Low","Middle", "High")),
             fill =  factor(SES_parent, levels = c("Low","Middle", "High")) ) ) +
    geom_errorbar(aes(ymin = asymp.LCL, ymax = asymp.UCL), width = 0.2,
                  position = position_dodge(width = 0.5)) +
    geom_line(position = position_dodge(width = 0.5)) +
    geom_point(size = 4, color = "black",
               position = position_dodge(width = 0.5)) +
    labs(x = "Time period", y = "Predicted dispersal probability",
```

```
          fill = "Parental SES",
          colour = "Parental SES",
          shape = "Parental SES") +
    ggtitle("Men") +
    theme_classic() +
    ylim(ymin = min(as.data.frame(ses.emm.scC)$asymp.LCL),
    ymax = max(as.data.frame(ses.emm.scC)$asymp.UCL) ) +
    scale_fill_manual( values = c("cyan3", "coral2","chartreuse2"))+
    scale_color_manual( values = c("cyan3", "coral2","chartreuse2")) +
    scale_x_discrete(labels = c("1760-1809", "1810-1899", "1900-1969"))+
    scale_shape_manual(  labels = c("Low", "Middle", "High"),
                         values = c(21,22,23))+
    theme(legend.position = "none",
           text = element_text(size = 18))
  ,
  ggplot(as.data.frame(ses.emm.scC) %>% filter(sex == "F"),
         aes(x = centuryCohort, y = prob,
             group = factor(SES_parent, levels = c("Low","Middle", "High")) ,
             shape = factor(SES_parent, levels = c("Low","Middle", "High")),
             colour = factor(SES_parent, levels = c("Low","Middle", "High")),
             fill =  factor(SES_parent, levels = c("Low","Middle", "High"))
         ) ) +
    geom_errorbar(aes(ymin = asymp.LCL, ymax = asymp.UCL), width = 0.2,
                  position = position_dodge(width = 0.5)) +
    geom_line(position = position_dodge(width = 0.5)) +
    geom_point(size = 4, color = "black",
               position = position_dodge(width = 0.5)) +

    labs(x = "Time period", y = "Predicted dispersal probability",
          fill = "Parental SES",
          colour = "Parental SES",
          shape = "Parental SES") +
    ggtitle("Women") +
    ylim(ymin = min(as.data.frame(ses.emm.scC)$asymp.LCL), ymax = max(as.data.frame(SES.emm.scC)$asy
mp.UCL) ) +
    theme_classic() +


    scale_fill_manual( labels = c("Low", "Middle", "High"),
                       values = c("cyan3", "coral2","chartreuse2"))+
    scale_color_manual( labels = c("Low", "Middle", "High"),
                        values = c("cyan3", "coral2","chartreuse2"))+
    scale_shape_manual(  labels = c("Low", "Middle", "High"),
                         values = c(21,22,23))+
```

```
    scale_x_discrete(labels = c("1760-1809", "1810-1899", "1900-1969")) +
    theme(legend.position = c(0.8,0.95),
          text = element_text(size = 18),
          legend.text = element_text(size = 16),
          legend.title = element_text(size = 16)
          #,legend.background = element_rect(fill = "white", color = "white")
    ) ,
  ncol = 2
)


#distance data
distdata <- SESdata %>%
  filter(dispersal == "yes" & !is.na(distanceMoved) & distanceMoved != 0 & centuryCohort != "1760-18
09")


#distance model
model_log <- lmer(log(distance) ~ sex*SES_parent*centuryCohort + (1 | mumid),
                  data = distdata )

Anova(model_log, type = 3)
summary(model_log)
#distance pairwise, emmeans
pairs( emmeans(model_log, ~ sex*centuryCohort*SES_parent, by = c("centuryCohort", "SES_parent"), typ
e = "response"),type = "response", reverse = T, adjust  = "none" )
pairs( emmeans(model_log, ~ sex*centuryCohort*SES_parent, by = c("sex", "SES_parent"), type = "respo
nse"),type = "response", reverse = T, adjust  = "none" )
pairs( emmeans(model_log, ~ sex*centuryCohort*SES_parent, by = c("centuryCohort", "sex"), type = "re
sponse"),type = "response", reverse = T, adjust  = "none" )


plotemm<- as.data.frame(emmeans(model_log, ~ sex*SES_parent*centuryCohort, type = "response") )
#plot distance
grid.arrange(
  ggplot(plotemm %>% filter(sex == "M"),
         aes(x = centuryCohort, y = response,
             group = factor(SES_parent, levels = c("Low","Middle", "High")) ,
             shape = factor(SES_parent, levels = c("Low","Middle", "High")),
             colour = factor(SES_parent, levels = c("Low","Middle", "High")),
             fill =  factor(SES_parent, levels = c("Low","Middle", "High"))
         )) +
    geom_errorbar(aes(ymin = asymp.LCL, ymax = asymp.UCL), width = 0.3,
                  position = position_dodge(width = 0.5)) +
    geom_line(linewidth  =1 , position = position_dodge(width = 0.5)) +
    geom_point(size = 4 ,  colour = "black",
               position = position_dodge(width = 0.5)) +
```

```
  labs(x = "Time period", y = "Predicted distance (km)",
       fill = "Parental SES",
       colour = "Parental SES",
       shape = "Parental SES") +
  ggtitle("Men") +
  theme_classic() +
  ylim(ymin = min(plotemm$asymp.LCL), ymax = max(plotemm$asymp.UCL) ) +
  theme(legend.position = "none",
        text = element_text(size = 18)) +
  scale_fill_manual( values = c("cyan3", "coral2","chartreuse2"))+
  scale_color_manual( values = c("cyan3", "coral2","chartreuse2")) +
  scale_shape_manual(  labels = c("Low", "Middle", "High"),
                     values = c(21,22,23))+
  scale_x_discrete(labels = c("1810-1899", "1900-1969"))

,

ggplot(plotemm %>% filter(sex == "F"),
       aes(x = centuryCohort, y = response,
           group = factor(SES_parent, levels = c("Low","Middle", "High")) ,
           shape = factor(SES_parent, levels = c("Low","Middle", "High")),
           colour = factor(SES_parent, levels = c("Low","Middle", "High")),
           fill =  factor(SES_parent, levels = c("Low","Middle", "High"))
       )) +
  geom_errorbar(aes(ymin = asymp.LCL, ymax = asymp.UCL), width = 0.3,
                position = position_dodge(width = 0.5)) +
  geom_line(linewidth  =1 ,position = position_dodge(width = 0.5)) +
  geom_point(size = 4 ,  colour = "black",
             position = position_dodge(width = 0.5)) +
  labs(x = "Time period", y = "Predicted distance (km)",
       fill = "Parental SES",
       colour = "Parental SES",
       shape = "Parental SES") +
  ggtitle("Women") +
  ylim(ymin = min(plotemm$asymp.LCL), ymax = max(plotemm$asymp.UCL) ) +
  theme_classic() +
  theme(legend.position = c(0.8,0.9),
        text = element_text(size = 18),
        legend.text = element_text(size = 16),
        legend.title = element_text(size = 16),
        legend.background = element_rect(fill = "white", color = "white"))+
  scale_fill_manual( labels = c("Low", "Middle", "High"),
```

```
                        values = c("cyan3", "coral2","chartreuse2"))+
    scale_color_manual( labels = c("Low", "Middle", "High"),
                         values = c("cyan3", "coral2","chartreuse2"))+
    scale_shape_manual(  labels = c("Low", "Middle", "High"),
                          values = c(21,22,23))+
    scale_x_discrete(labels = c("1810-1899", "1900-1969")),
  ncol = 2
)
```
